# Effect of Superheated Steam Treatment on Rice Quality, Structure, and Physicochemical Properties of Starch

**DOI:** 10.3390/foods14040626

**Published:** 2025-02-13

**Authors:** Ziyu Wang, Ziwei Xiao, Jing Ye, Juan Li, Xinxia Zhang, Ting Li, Li Wang

**Affiliations:** 1 State Key Laboratory of Food Science and Resources, School of Food Science and Technology, Jiangnan University, Lihu Road 1800, Wuxi 214122, China; 2 National Engineering Research Center for Cereal Fermentation and Food Biomanufacturing, Jiangnan University, Lihu Road 1800, Wuxi 214122, China; 3 Academy of Jiangsu Grain Science and Technology Innovation, Nanjing 210003, China; 4 Jiangsu Provincial Engineering Research Center for Bioactive Product Processing, Jiangnan University, Lihu Road 1800, Wuxi 214122, China; 5 Key Laboratory of Carbohydrate Chemistry and Biotechnology, Ministry of Education, Jiangnan University, Lihu Road 1800, Wuxi 214122, China

**Keywords:** superheated steam, rice starch, structural changes, rice quality, rheological properties

## Abstract

This study aimed to investigate the effect of superheated steam treatment on the cooking and eating quality of rice, and further explore the effect of superheated steam treatment on the structure, gel properties, and rheological behavior of rice starch. After superheated steam treatment, the optimal cooking time of rice was effectively reduced by 26.9%, and the taste value of rice was significantly improved, from 78.45 to 84.20, when treated at 155 °C for 10 s. Superheated steam treatment significantly reduced the amylose and protein content, and increased the average particle size of rice starch. Compared with the control, the enthalpy change (ΔH) in the superheated steam treatment rice starch decreased notably from 6.53 to 5.28 after treatment, the relative crystallinity of the starch was significantly reduced from 21.20 to 10.89, and the short-term order of the starch was enhanced owing to the rearrangement of starch molecules after gelatinization. The starch structure was more compact and orderly after the superheated steam treatment, which significantly improved the hardness, viscoelasticity, and strength of the gel. These results indicate that superheated steam treatment improves the quality of rice by changing the structure of rice starch.

## 1. Introduction

Rice is one of the most widely cultivated cereal crops in the world, with over 65% of the population in China relying on rice as their staple food [1]. Starch is the main component of rice, accounting for about 85% of its dry matter, and it has a unique structure and physicochemical properties [2]. The structure and composition of rice starch play significant roles in influencing the quality of rice [3]. At present, rich starch foods are mainly modified by physical, chemical, and enzyme modification to meet the needs of improvement in the quality of rice. However, chemical modification has residual chemical reagent components that affect food safety, and the cost of enzyme modification is expensive. Therefore, physical modifications that are environmentally friendly during processing have received widespread attention in the field of food technology [4].

Recently, applying superheated steam technology in the food industry has been gaining popularity as an innovative technology that can dry, sterilize, inactivate, and modify enzymes [5]. The superheated steam treatment (SST) had significant inhibitory and killing effects on bacillus and mold in wheat grains [6]. Wang et al. effectively reduced the lipase activity and peroxide activity of barley by 38.85% and 100%, respectively, after heating barley at 160 °C for 6 min [7]. Hu et al. [8] found that SST can significantly shorten the cooking duration and overall hardness. Chen et al. [9] reported that flour treated with superheated steam enhanced its water-holding capacity, widened the gelatinization temperature range, and improved the overall structural stability. Meanwhile, SST significantly affected the structure and functional characteristics of grain starch, ultimately leading to improved processing quality. Almeida et al. [10] studied the extraction of rice starch using superheated steam treatment and found that superheated steam modified the structure and morphology of starch, resulting in properties different from natural starch. Liu et al. [11] used superheated steam to directly treat wheat grains. Due to the changes in starch granule and molecular structure, the physicochemical properties changed, improving the specific volume and leading to a texture like cake. Kim et al. [12] reported that SST reduced the crystallinity and structural orderliness of rice starch, which was related to significant reductions in cooking time and grain hardness. These results indicate that superheated steam is a promising physical method for improving rice characteristics. However, the research on the modification mechanism of rice starch by superheated steam treatment is mostly based on the SS treatment of rice flour or rice starch. Therefore, the changes in starch structure and properties in rice grains treated with superheated steam are still unclear.

In recent years, much research has effectively improved the quality of rice through superheated steam. In previous studies, we extracted protein from SST rice and found that SST led to protein aggregation and a more ordered secondary structure. Simultaneously, a possible explanation for the impact of SST on the gelatinization characteristics and taste quality of rice from a protein perspective was provided [13]. However, the current research on the treatment of rice grains with superheated steam and its impact on rice starch is not very in depth. Therefore, this study used superheated steam at different temperatures and durations for rice pretreatment, observing changes in rice cooking quality and taste value. In addition, we explored the impact of SST on rice grain quality from the perspective of starch structure and physicochemical properties. Overall, this research contributes to providing an effective technology (SST) for improving the quality of rice.

## 2. Materials and Methods

### 2.1. Materials

Commercial Akita Komachi rice was purchased from COFCO Fulinmen Co., Ltd. (Beijing, China). Sodium hydroxide, concentrated sulfuric acid, hydrochloric acid, and other chemicals and reagents were purchased from Sinopharm Chemical Co., Ltd. (Shanghai, China).

### 2.2. Sample Preparation

The rice samples were prepared by using SST equipment (model WS-FMD15, Jiangsu Wanchuang Sterilization Equipment Technology Co., Ltd., Zhenjiang, China). The temperature of the overheated air was adjusted to 155 °C, 170 °C, and 185 °C. After preheating the processing room, 1000 g of rice were added to the feeding system and stirred at high speed using a steam mixer. The process parameters were as follows: 155 °C for 5 s (AK-155-5), 10 s (AK-155-10), 15 s (AK-155-15), and 20 s (AK-155-20), and 5 s at 170 °C (AK-170-5) and 185 °C (AK-185-5). The rice that had not been treated with SST was the control sample (AK). Then, the rice grains were put into sterile valve bags and stored at 4 °C in the refrigerator.

### 2.3. Preparation of Starch

Rice grains were grinded with a high-speed grinder and screened through a 100-mesh screen to obtain rice flour. The rice starch was separated by the method of Li et al. [14], with appropriate modifications. Firstly, a certain amount of rice flour was soaked in distilled water for 12 h. Then, the rice flour was soaked in a 0.2% NaOH solution in a ratio of 1:8 and stirred for 12 h. Afterward, the starch solution was centrifuged at 4000 rpm for 10 min, and the supernatant and impurities were discarded. Finally, the obtained white sediment was resuspended with distilled water. The pH value was adjusted to 7.0 with dilute hydrochloric acid before being centrifuged again. The starch layer was washed three times with distilled water. Then, the obtained white starch was freeze-dried and crushed through a 100-mesh sieve to obtain rice starch, and it was stored at 20 °C.

### 2.4. Chemical Composition Analysis

The moisture content of the sample was determined by direct drying in an oven until the weight remained constant [15]. The Kjeldahl method [16] was used to determine protein content. The method described by Juliano [17] was used to evaluate the amylose content of rice samples, with partial modifications.

### 2.5. Taste Value, Optimal Cooking Time, and Sensory Evaluation

The taste value of the rice was measured using the STA1B device (Satake Co., Ltd., Hiroshima, Japan). A certain amount of rice in grams was placed in a dedicated cup. The mass ratio of rice to water was 1:1.4. After soaking for 30 min, steaming for 30 min, and simmering for 10 min, the rice was cooled for 2 h. Subsequently, a rice cake was pressed using 8.0 g of cooked rice for further measurement.

The cooking time of the rice samples was determined using the glass plate–white center method [18]. First, a 5 g rice sample was added to 50 mL of boiling water and heated, and then the cooking time was immediately measured. After cooking for 20 min and every minute thereafter, 10 grains of rice were taken out and pressed with a glass plate against a black background. When the rice grains did not contain a white core, the cooking time was recorded and cooking was stopped.

The rice was cooked according to the method of taste value. Each sample was evaluated 3 times, and the average score was measured for the final score. The sensory group (20 members) identified various sensory indicators of rice in the sensory testing laboratory. The sensory attributes and scoring criteria of rice are shown in Table S1.

### 2.6. Scanning Electron Microscopy (SEM)

The morphological structure of untreated and SST rice starch was observed using a scanning electron microscope (SEM; S-4700, Hitachi, Tokyo, Japan) with an acceleration voltage of 3.0 kV and magnifications of 1000 and 2000 times. Before SEM observation, conductive materials were used to fix the starch sample on the stage, and it was sprayed with a layer of gold.

### 2.7. Particle Size Measurement

The particle size distribution of the rice starch was determined by laser particle size analyzer. A certain concentration of rice starch suspension was prepared, and then a reasonable amount of sample was added dropwise to the instrument. The test opacity was between 5% and 10%. D10, D50, and D90 represent the particle sizes with 10%, 50%, and 90% cumulative distribution, respectively. D10, D50, D90, the volume average diameter D(4,3), and the area average diameter D(3,2) of the sample were recorded.

### 2.8. Differential Scanning Calorimetry (DSC)

The thermodynamic properties of the rice starch were assessed by differential scanning calorimetry (DSC3+, Mettler Toledo, Zurich, Switzerland). The sample was weighed in an aluminum crucible, and then distilled water was added to adjust the sample water ratio to 1:3 (g/g). Before analysis, the pan was sealed and balanced at 4 °C for 12 h. The program was set to a starting temperature of 30 °C, an ending temperature of 100 °C, and a heating rate of 10 °C/min, and the starting temperature (T_o_), peak temperature (T_p_), ending temperature (T_c_), and enthalpy value (ΔH) were recorded.

### 2.9. X-Ray Diffraction

The crystal structure and relative crystallinity of the rice starch were measured using an X-ray diffractometer (D2 PHASER, Bruker, Germany). The operating conditions were as follows: The samples were scanned at 5–40° (2θ) at a rate of 2°/min, a target voltage of 40 kV, a step size of 0.03°, and a current of 30 mA. Crystallinity calculation was performed using MDI Jade 6.0 software.

### 2.10. Fourier Transform Infrared Spectroscopy (FTIR)

The short-range ordered structure of the rice starch was determined by a Fourier transform infrared spectrometer (IS10, Nicolet, Madison, WI, USA). The rice starch samples were mixed with KBr in a ratio of 1:100 (*w*/*w*) and ground thoroughly in a mortar until uniform. The powder was pressed into a transparent sheet. Then, the spectral data were collected by scanning the sample 30 times at a resolution of 4 cm^−1^ within the range of 4000–400 cm^−1^. The short-range order parameter was obtained by counting the ratio of the peak intensities at 1047 cm^−1^ and 1022 cm^−1^ using OMNIC 8.2 software.

### 2.11. Small-Angle X-Ray Scattering (SAXS)

The layered structure of the starch samples was analyzed using a small-angle X-ray scattering spectrometer (SAXS; SAXSpoint2.0, Anton Paar Company, Graz, Austria), following the method described by Ma et al. [19]. The appropriate amount of starch sample was mixed thoroughly with distilled water of equal weight. Then, the mixture was left at 4 °C for 12 h. The prepared starch slurry was used for SAXS analysis, with a pure water sample as the blank. The specific equipment parameters were set as follows: the operating voltage of the X-ray source was 50 kV, the power was 30 W, and the wavelength of Cu K α rays was 1.5418 Å. DIFFRAC plus Nano Fit software was used to process the data. The calculation formula for the average thickness (D) of the semi-crystalline layer is shown in the following equation:

(1) $$D=\frac{2\pi }{I_{\max}}$$

where *I_max_* is the value corresponding to the peak position at 0.6 nm^−1^ in the SAXS spectrum.

### 2.12. Texture Profile Analysis (TPA)

The gel characteristics of the rice starch were analyzed by texture analyzer (TA. XT. plus, Texture Technologies Corp., Scarsdale, NY, USA). The hardness, elasticity, and other texture characteristics of the rice starch gel were obtained though TPA. A certain amount of sample was prepared in a 12% starch suspension using water. The starch suspension was heated at a 95 °C constant-temperature water bath, then cooled at 25 °C and stored at 4 °C for 12 h. The starch gel was then placed on the target platform and compressed with a probe with a diameter of 25 mm. The pre-test, test, and post-test speeds were set at 1.0 mm/s. The compression distance was 50%, the 2 compression intervals were 5.0 s, and the trigger force was 5.0 g.

### 2.13. Rheological Properties

The rheological behavior of the rice starch was determined using the method described by Gong et al. [20]. A certain concentration of rice starch paste sample was placed on the rheometer testing platform and subjected to rheological measurements using a 40 mm flat fixture. The sample was equilibrated for 180 s to eliminate the mechanical effects caused by loading the sample. The gap between the testing platform and the flat fixture was set to 1000 μm. To determine the linear viscoelastic zone, an amplitude scanning test was conducted within the strain range of 0.1% to 10%, the temperature was set to 25 °C, and the angular frequency was set to 1 rad/s. A dynamic frequency scanning test was conducted at temperature of 25 °C and a strain of 1% (obtained from amplitude scanning test results) within the angular frequency range of 0.1~100 rad/s. The curves of the storage modulus (G′) and loss modulus (G″), and the loss tangent (tan δ = G″/G′), were recorded as functions of frequency.

### 2.14. Statistical Analysis

Origin 2018 software (Origin Lab Corp., Northampton, MA, USA) was used to plot the experimental data. The statistical analysis was conducted using SPSS (version26.0; SPSS Inc., Chicago, IL, USA). The differences between samples were analyzed by Duncan’s multiple-comparison test, and there was a significant difference in *p* < 0.05.

## 3. Results and Discussion

### 3.1. Chemical Composition

The chemical composition of the SST rice was analyzed and compared with the AK (as shown in Table 1). The moisture content of the SST rice under different conditions showed different patterns of change. The results showed that at 155 °C, the moisture content of the treated rice first increased and then decreased with the extension of treatment time, reaching its maximum value at 10 s. At a processing time of 5 s, the moisture content of the rice treated with superheated steam showed a trend of first increasing and then decreasing, reaching its highest point at 170 °C. This is due to the unique initial condensation phenomenon of superheated steam. At the initial stage of processing, the surface of the rice samples condensed steam, and the moisture content briefly increased, which is consistent with the experimental results of Wu et al. [21].

The amylose content of AK-155-5, AK-155-10, AK-155-15, and AK-155-20 was 19.9%, 19.83%, 17.37%, and 16.65%, respectively. At 155 °C, the content of amylose decreased with the prolongation of treatment time, and the content of amylose significantly decreased after 15 s of treatment. When the processing time was fixed at 5 s, the content of amylose decreased with increasing temperature. The decrease in amylose content is mainly due to the degradation of starch chains and the destruction of their double-helix structures [22]. When the temperature exceeds a certain threshold, heat treatment triggers internal changes in starch molecules, which may lead to glycosidic bond breakage in amylopectin and even amylose. Glycoside bond breakage results in excessive degradation of starch molecules and a decrease in starch chain length [23]. This change is contrary to the research results of Li et al. [11] on the proportion of SST to wheat amylose content, indicating that the effect of SST may be affected by grain varieties and amylose content.

Under the condition of 155 °C, the protein content of rice treated with superheated steam initially decreased and then increased, reaching the lowest value of 5.8% at 10 s. At a processing time of 5 s, as the temperature increased, the protein content of the treated rice showed a similar trend.

### 3.2. Taste Value, Optimal Cooking Time, and Sensory Evaluation

**Figure 1:**
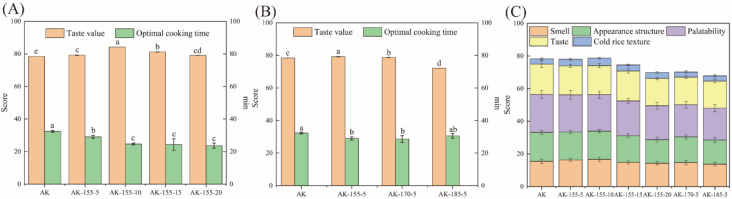
The taste value, optimal cooking time (**A**,**B**), and sensory evaluation (**C**) of untreated and SST rice. (**A**) Untreated rice and rice treated with SST at 155 °C for 5, 10, 15, and 20 s; (**B**) untreated rice and rice treated with SST at 155, 170, and 185 °C for 5 s. There is a significant difference (*p* < 0.05) in the values marked with different lowercase letters in each subgraph.

The changes in taste value and cooking time of the rice are shown in Figure 1. SST effectively shortened the optimal cooking time of rice from 32.3 min to 23.6 min, ranging from 1.7 to 8.7 min. This reduction was in accordance with the results of Piyawanitpong et al. [24], where SST showed an increase in the porous structure of the rice grains, thereby improving water absorption and shortening cooking time. In our previous study, the water absorption rate of SST rice increased from 343.22% to 467.59%, while causing a significant increased volume expansion rate in the rice from 5.06% to 8.89% [13]. As shown in Figure 1, the taste value of Ak-155-10 was distinctly improved compared with that of AK, and a suitable SST could effectively improve the sensory score of the rice. However, when overprocessed, the AK-185-5 rice score decreased, which may have been due to the aging of the rice caused by high temperatures. The quality of the rice was influenced by various factors. High protein content can strengthen the protein–starch interaction, affect the water absorption and gelatinization process of starch, strengthen the starch gel network, and thus increase the hardness of the rice [25]. Ma et al. [26] also pointed out that cooked rice with a high amylose content had a harder texture. Therefore, the increase in taste value after SST may be due to the decrease in protein and amylose content in rice.

The changes in the sensory evaluation of the rice are shown in Figure 1. SST had different effects on the smell, appearance structure, palatability, taste, and cold texture of the rice. The smell score of cooked rice initially increased and then decreased. Low temperature and short duration of SST caused the rice to produce a taste that consumers liked. The increase in SST temperature and time resulted in a decrease in the appearance score of the rice, which was possibly due to the rice becoming more yellow in color. SST improved the texture of cold rice, which may also have helped enhance the taste of the SST rice.

### 3.3. Morphology of Rice Starch Granules

**Figure 2:**
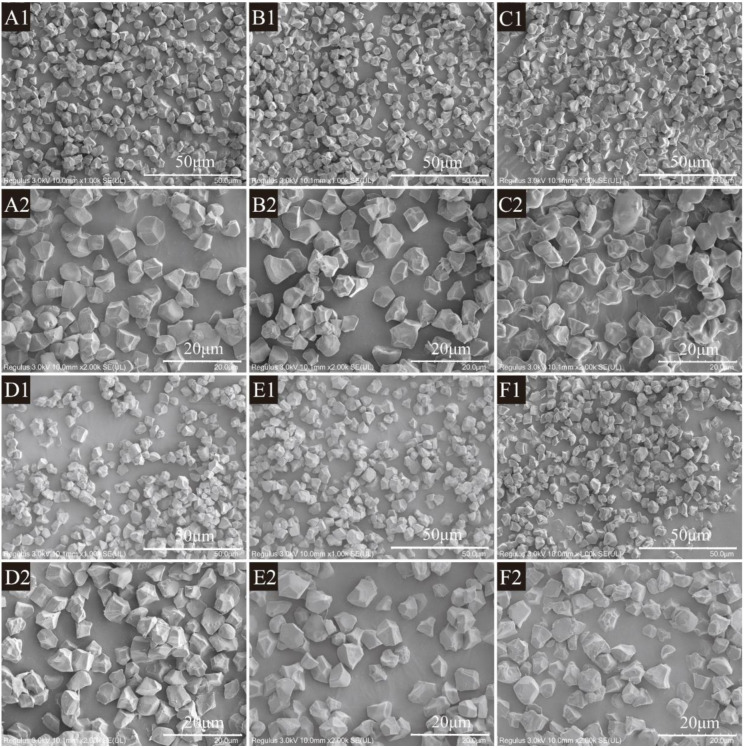
Granular structure of untreated and SST rice starch at × 1000: (**A1**~**F1**); at × 2000: (**A2**~**F2**). (**A1**,**A2**): AK; (**B1**,**B2**): AK-155-10; (**C1**,**C2**): AK-155-20; (**D1**,**D2**): AK-155-5; (**E1**,**E2**): AK-170-5; (**F1**,**F2**): AK-185-5.

The microstructure of starch granules observed by SEM is shown in Figure 2. The granule morphology of the AK starch samples demonstrated polygonal or irregular geometry, with distinct edges and corners, and a relatively smooth surface. The microstructure of the SST rice starch was similar to that of the untreated rice, with the starch maintained its granular structure intact. After being treated with superheated steam, the rice starch granules showed significant aggregation, and the degree of crushing and the degree of dispersion were reduced. This change can be attributed to the gelatinization of the starch. In addition, the high temperature also promoted hydrogen bonding of the starch molecules [27]. The effect of treatment time on starch morphology was greater than that of temperature. Especially under the treatment condition of 170 °C and 20 s, the surface of the particles appeared concave, which may have been due to the gelatinization and aggregation of the starch particles during the superheated steam treatment process. At the same time, the irregular edges became blurred and smooth, indicating that starch was degraded and recrystallized.

### 3.4. Particle Size Distribution of Rice Starch

**Figure 3:**
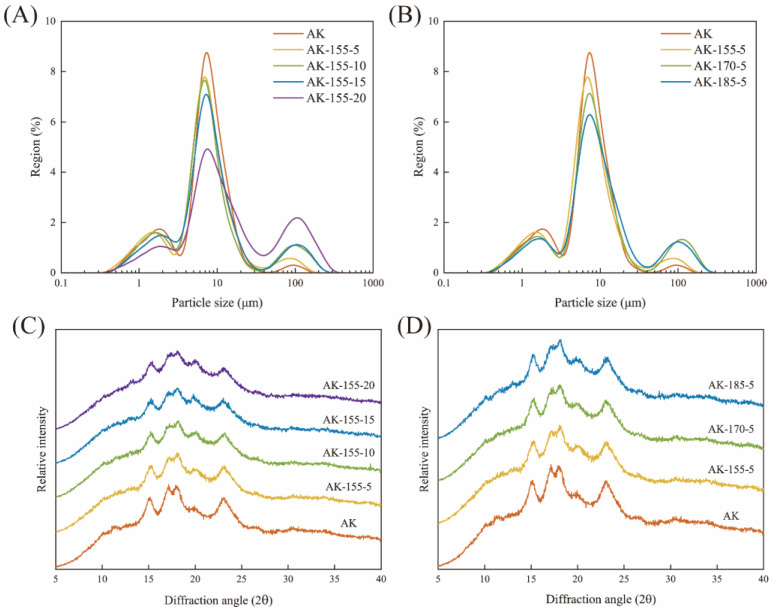
Particle size distribution (**A**,**B**) and X-ray diffraction patterns (**C**,**D**) of untreated and SST rice starch. (**A**,**C**) At different times; (**B**,**D**) at different temperatures.

The particle size distribution of the rice starch is shown in Figure 3 and Table 2. All starch samples showed a trimodal distribution. As the processing time increased, the peak height at 10 μm gradually decreased, while the peak height at 100 μm gradually increased, and D(4,3) significantly increased from 9.35 μm to 36.23 μm. This indicated that superheated steam promoted the expansion of the starch particles, which is consistent with the phenomenon of particle morphology (Figure 2). Hu et al. [6] also observed that when wheat was treated with superheated steam, the flour particle size gradually increased with the treatment temperature and time. SST causes aggregation effects between rice starch particles, while high-temperature treatment may lead to partial gelatinization and adhesion of starch, causing starch structure expansion [28]. The larger particle size was conducive to enhancing the effective combination of starch particles and water, promoting the full expansion of the particles and thus enhancing the water-holding capacity and viscosity of starch gel.

### 3.5. DSC

The thermodynamic properties of untreated and SST rice starch are presented in Table 3. During starch gelatinization, the highly ordered crystalline phase within the starch molecules dissociates, transitioning into an amorphous structure. After SST, the onset temperature (T_o_) increased, while the peak temperature (T_p_) and conclusion temperature (T_c_) decreased significantly. Concurrently, as the processing time and temperature increased, the enthalpy change in the SST rice starch decreased notably, suggesting that the gelatinization process during SST disrupts the double-helix structure of the starch, leading to its gelatinization. The gelatinization range (T_o_–T_c_) of starch exhibited a declining trend, indicating the homogenization of different microcrystals [29]. The increase in the thermal phase transition temperature, coupled with the X-ray diffraction results discussed in Section 3.6, suggests that the partially unglazed starch structure became more stable and ordered [30].

### 3.6. Long-Range Crystal Structure of Rice Starch

The X-ray diffraction pattern of the starch is shown in Figure 3, and the crystallinity is shown in Table 3. All starch samples exhibited similar crystal structures, with two single peaks at 15° and 23° and one double peak at 17° and 18°. The characteristic peaks of SST starch did not show any shift or change in position, indicating that SST did not alter the A-type crystal structure of starch. This is consistent with the research of Xiao et al. [31] on the heat–moisture treatment of buckwheat starch. The weak small peak appearing around 20° indicated that the rice contained a small amount of starch lipid complexes, forming a V-shaped crystalline structure [32].

The relative crystallinity of the SST rice starch decreased from 21.2% to 10.89%. Superheated steam treatment caused the hydrogen bonding network of the starch molecules to be disrupted, leading to the breakdown of the helical structure and subsequently affecting the integrity of the crystallization region [33,34]. The reduction in relative crystallinity was also related the enlargement in the starch particle size and the decrease in amylose content. When the processing time was fixed at 5 s, the crystallinity of the SST starch decreased slightly with the increase in temperature after 170 °C. This showed that the extension of processing time had a more significant impact on the crystallinity of the starch than the increase in processing temperature. This trend is also reflected in the research results of Raphael et al. [10].

### 3.7. Short-Range Ordered Structure of Rice Starch

**Figure 4:**
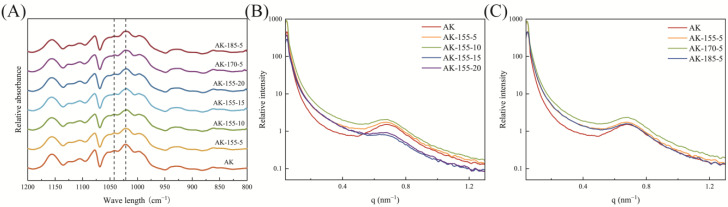
Fourier infrared spectra (**A**) and layered structure (**B**,**C**) of untreated and SST rice starch. (**B**) At different times; (**C**) at different temperatures.

FTIR can reflect the changes in the short-range ordered structure of starch granules [35]. As shown in Figure 4, the spectral characteristics of the AK and SST rice starch were consistent, indicating that the structure of functional groups in the rice starch before and after SST did not change; that is, superheated steam induced a physical modification process. This is consistent with the XRD test results in Figure 3. The crystal structure of the starch was closely related to the absorbance at 1047 cm^−1^ and 1022 cm^−1^ [36]. The measured 1047 cm^−1^/1022 cm^−1^ is shown in Table 3. The ratio of SST rice starch was higher than that of untreated samples (the ratio was 0.758), indicating that superheated steam treatment can improve the short-range order structure of starch. As the processing time increased, the value of 1047 cm^−1^/1022 cm^−1^ showed a trend of first increasing to 0.798 (AK-155-10) and then decreasing to 0.770 (AK-155-20). At a processing time of 5 s, as the temperature increased, the order structure of SST rice starch also showed an upward trend. The starch structure of rice treated with superheated steam was more ordered, possibly due to the annealing effect [37]. When starch is heated and reaches its glass transition temperature, the fluidity of starch chains increases and the unstable structure at the short-range scale is disrupted, resulting in a rearrangement of starch chains [7]. During SST, the water provided not only promotes the gelatinization and degradation of starch, but also promotes the self-assembly of starch chains, leading to the entanglement of amylopectin [38]. Pressurizing and heating may make the starch granule structure compact [39]. The results were different from the trend of a decreasing short-range ordered structure of potato starch and wheat flour in SST, and an increasing short-range ordered structure of black rice in SST [8,40,41], which emphasizes the different thermal responses of starch in different cereal varieties.

### 3.8. Layered Structure of Rice Starch

SAXS was used to further investigate the layered structure of starch granules. Figure 4 shows the SAXS pattern of the rice starch. The SAXS spectra of all starch samples showed a layered structure at the 0.69 nm^−1^ position, which was formed by the periodic alternating arrangement of crystalline and amorphous layers of amylopectin. The relative intensity of the layered peak of the rice starch treated with superheated steam showed a pattern of first increasing and then decreasing as treatment time and temperature increased. The increase in relative intensity of the layered starch peaks indicates that the crystalline region of the starch granules had a tighter double-helix structure. The change in peak intensity of SAXS during treatment at 155 °C confirmed the results of FTIR in Section 3.7, which showed that the double-helix structure in the crystalline region became neatly arranged, and the most ordered structure occurred at 155 °C and 10 s. SST affected the layered structure of rice starch, making the ordered arrangement of semi-crystalline growth rings more regular and improving the orderliness of starch. The data details of the thickness of the semi-crystalline layer are shown in Table 3, with interlayer spacing ranging from 9.12 to 9.19 nm. As the processing time increased and the processing temperature rose, the thickness of the semi-crystalline layer first decreased and then increased. The change in the layered structure of the starch samples may be related to partial gelatinization of starch granules and the relative increase in particle size. In the study by Ma et al. [18], SST promoted the transition of a wheat starch layered structure from crystalline flakes to amorphous flakes. During SST, the hydrogen bonds that maintain the integrity of crystal flakes are broken in a high-temperature environment with limited moisture, and the starch particles connected by hydrogen bonds are destroyed, resulting in the dissolution of the flake structure [18].

### 3.9. Gel Properties of Rice Starch

The texture attributes of the SST rice starch gel, such as hardness, adhesion, cohesion, and viscosity, are shown in Table 4. SST increased the hardness, adhesiveness, chewiness, and adhesive of the starch gel, indicating that the extrusion treatment was helpful to form a uniform network structure in the process of recrystallization. At 155 °C, with the increase in treatment time, the hardness of the rice starch gel increased and then diminished, reaching the highest value of 323.13 g at 10 s. The adhesiveness significantly increased, while the elasticity, cohesion, adhesive, and chewiness all increased first and then decreased. Amylose molecules could improve the thermal stability of the starch crystals, limit starch expansion, and make the texture harder. The phenomenon of decreased amylose content but increased hardness in the SST sample may have been due to the denser and more ordered structure of the partially unglazed starch in the SST rice. Due to intermolecular interactions, amylopectin makes starch particles stronger, resulting in a more elastic and harder texture. Another possible explanation is that the recombination and interaction between non-starch and starch components are induced by SST [42]. When the treatment time was fixed at 5 s and the temperature was raised to 170 °C or above, the SST had no remarkable influence on the hardness, adhesiveness, or chewiness of the starch gel. In general, superheated steam treatment can improve the gel properties of rice starch, especially at 155 °C for 10 s. With the increase in time and temperature, the viscosity of rice starch gel gradually increased, which was due to the leaching of amylose during gelatinization and the increase in particle size.

### 3.10. Rheological Behavior of Rice Starch

**Figure 5:**
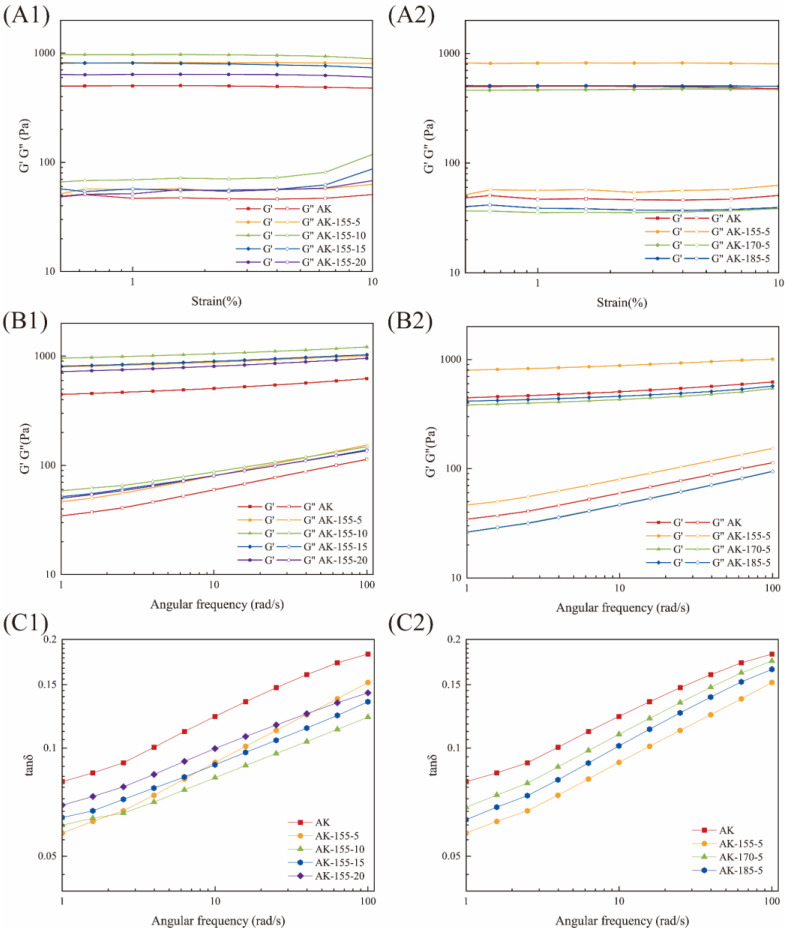
Rheological properties of untreated and SST rice. (**A1**,**A2**) Strain scans; (**B1**,**B2**) the elastic modulus and viscous modulus of dynamic frequency scanning; (**C1**,**C2**) the loss tangent of dynamic frequency scanning.

The amplitude scanning results of the AK and SST rice are shown in Figure 5. The linear viscoelastic domain (LVR) was determined to be within the stress range of 0.5~4% through amplitude scanning testing, and the 1% strain was selected for frequency scanning testing in the LVR. The values of G′ and G″ in LVR revealed the elasticity and viscosity of the gel, respectively. SST significantly increased the difference between G′ and G″ in the LVR of the rice starch gel, and G′ was greater than G″, indicating that the rice starch gel was closer to the solid state after SST [43]. At the same time, the highest G′ value occurred after treatment at 155 °C for 10 s, indicating that the stability of the SST starch sample had been improved and was not easily damaged. The critical strain of the rice starch gel decreased, indicating that SST weakened the deformability of the starch gel.

The dynamic frequency scanning results of the untreated and SST rice starch are shown in Figure 5. Both G′ and G″ increased with the increase in angular frequency, which indicated that starch gel was a weak gel [44]. The changes in G′ were more pronounced than those in G″, indicating that the increase in elastic properties was more significant than that in viscous properties. After SST at 155 °C, the G′ and G″ of the SST samples were higher than those of AK, indicating that the gel strength of the treated rice starch was enhanced. Yang et al. [45] proposed that this may be due to the degradation of starch molecular chains caused by hydrothermal treatment, which is conducive to the rearrangement of starch molecules, making starch molecules form a continuous gel network structure and thus enhancing its gel strength. As the processing time increased, both the G′ and the G″ value presented first an increasing and then a decreasing change, and the change in the elastic modulus was consistent with the previous measurement results for texture properties. The change trend of the G″ value was different from that of viscoelasticity. The viscosity of the rice starch gel gradually increased, mainly due to the leaching of amylose during gelatinization. The G′ and G″ values of AK-170-5 and AK-185-5 were both lower than the corresponding values of AK, which may have been due to excessive melting of the crystals.

The change in tanδ can be used to reflect the viscoelasticity of the starch samples. The tanδ of the untreated and SST rice was less than 1. This means that the rice noodle gel was more inclined to form elastic morphology and had higher gel strength, which may be attributable to the formation of a weak gel system. After SST, the tanδ value of the starch gel decreased, so the contribution of elastic components increased relatively after SST.

## 4. Conclusions

The results showed that the superheated steam treatment significantly affected the particle size distribution, microstructure, and gelation behavior of the rice starch. It also reduced the amylose content and protein content of the rice. In addition, the starch particles showed higher swelling and larger particle size, which effectively improved the cooking time and taste value of the rice. The superheated steam treatment significantly reduced the ΔH and the relative crystallinity of the starch, which may have been due to the partial gelatinization of the starch. In the superheated steam-treated rice, the formation of an ordered structure took place, and the short-range ordering of starch was enhanced, which proved that the starch structure was more compact. SST made the crystal growth ring of the rice starch more regular than that of the untreated rice. This improved the elasticity and hardness of the starch gel, and the elasticity and hardness reached their maximum when processed at 155 °C for 10 s. In terms of rheological properties, the G′ and G″ of the SST rice starch increased, and the gel strength was enhanced. At the same time, the increase in elastic properties was more obvious than that of the viscosity properties, and the gel tended to be solid. In conclusion, superheated steam treatment improved the quality of the rice by changing the internal structure of the starch. The application effect of superheated steam treatment at 155 °C for 10 s was the best.

## Figures and Tables

**Table 1:** Chemical composition of rice flour.

Sample	Moisture (%)	Protein (%)	Amylose (%)
AK	12.58 ± 0.04 ^d^	6.64 ± 0.03 ^a^	20.14 ± 0.22 ^a^
AK-155-5	13.41 ± 0.03 ^c^	6.36 ± 0.07 ^c^	19.90 ± 0.11 ^a^
AK-155-10	13.92 ± 0.05 ^a^	5.80 ± 0.05 ^e^	19.83 ± 0.00 ^a^
AK-155-15	13.75 ± 0.05 ^b^	6.02 ± 0.02 ^d^	17.37 ± 0.34 ^c^
AK-155-20	13.73 ± 0.00 ^b^	6.44 ± 0.01 ^b^	16.65 ± 0.00 ^d^
AK-170-5	13.69 ± 0.04 ^b^	6.45 ± 0.01 ^b^	19.11 ± 0.34 ^b^
AK-185-5	12.56 ± 0.04 ^a^	6.57 ± 0.04 ^a^	18.87 ± 0.22 ^b^

The values labeled with different lowercase letters in each column have significant differences (*p* < 0.05).

**Table 2:** Particle size distribution of untreated and SST rice starch.

	AK	AK-155-5	AK-155-10	AK-155-15	AK-155-20	AK-170-5	AK-185-5
D10 (μm)	1.53 ± 0.01 ^e^	1.42 ± 0.02 ^f^	1.57 ± 0.02 ^d^	1.74 ± 0.00 ^b^	2.29 ± 0.01 ^a^	1.59 ± 0.01 ^d^	1.70 ± 0.01 ^c^
D50 (μm)	7.15 ± 0.02 ^de^	6.83 ± 0.04 ^e^	7.08 ± 0.76 ^de^	7.44 ± 0.12 ^cd^	10.17 ± 0.37 ^a^	7.78 ± 0.05 ^bc^	8.06 ± 0.96 ^b^
D90 (μm)	14.59 ± 0.52 ^e^	18.00 ± 1.56 ^e^	57.83 ± 2.98 ^d^	62.30 ± 0.65 ^d^	119.70 ± 1.98 ^a^	81.31 ± 4.27 ^b^	70.24 ± 0.66 ^d^
D(4,3) (μm)	9.35 ± 0.08 ^e^	12.03 ± 0.98 ^e^	18.09 ± 0.04 ^d^	18.60 ± 0.09 ^d^	36.23 ± 0.21 ^a^	22.18 ± 1.49 ^b^	20.68 ± 0.01 ^c^
D(3,2) (μm)	3.93 ± 0.00 ^e^	3.78 ± 0.06 ^f^	4.12 ± 0.07 ^d^	4.38 ± 0.03 ^c^	5.71 ± 0.00 ^a^	4.44 ± 0.02 ^c^	4.53 ± 0.03 ^b^

The values marked with different lowercase letters in each line have significant differences (*p* < 0.05).

**Table 3:** Thermal characteristic parameters, relative crystallinity, short-range order, and semicrystalline layer thickness of untreated and SST rice starch.

	AK	AK-155-5	AK-155-10	AK-155-15	AK-155-20	AK-170-5	AK-185-5
T_o_ (°C)	56.86 ± 0.16 ^ab^	57.25 ± 0.65 ^a^	56.26 ± 0.12 ^b^	56.93 ± 0.16 ^ab^	56.62 ± 0.06a ^b^	57.26 ± 0.21 ^a^	57.10 ± 0.21 ^a^
T_p_ (°C)	64.21 ± 0.13 ^a^	63.63 ± 0.03 ^b^	62.61 ± 0.01 ^c^	62.53 ± 0.13 ^c^	62.75 ± 0.23 ^c^	63.37 ± 0.12 ^b^	63.54 ± 0.12 ^b^
T_c_ (°C)	70.19 ± 0.11 ^a^	69.44 ± 0.21 ^b^	68.39 ± 0.22 ^de^	68.09 ± 0.25 ^e^	68.51 ± 0.13 ^cde^	68.74 ± 0.27 ^cd^	68.9 ± 0.10 ^c^
ΔH	6.53 ± 0.06 ^a^	5.88 ± 0.17 ^b^	5.61 ± 0.04 ^bc^	5.46 ± 0.15 ^cd^	5.28 ± 0.05 ^d^	5.82 ± 0.19 ^b^	6.25 ± 0.06 ^a^
Relative crystallinity (%)	21.20 ^a^	16.68 ^b^	14.46 ^c^	12.28 ^f^	10.89 ^g^	13.18 ^d^	13.00 ^e^
1047 cm^−1^/1022 cm^−1^	0.758 ± 0.00 ^e^	0.783 ± 0.00 ^c^	0.798 ± 0.01 ^a^	0.789 ± 0.02 ^bc^	0.770 ± 0.00 ^d^	0.787 ± 0.01 ^bc^	0.794 ± 0.00 ^ab^
D (nm)	9.17 ^c^	9.15 ^d^	9.12 ^f^	9.18 ^b^	9.19 ^a^	9.13 ^e^	9.15 ^d^

The values marked with different lowercase letters in each line have significant differences (*p* < 0.05).

**Table 4:** Gel properties of untreated and superheated steam-treated rice starch.

	AK	AK-155-5	AK-155-10	AK-155-15	AK-155-20	AK-170-5	AK-185-5
Hardness (g)	185.48 ± 2.59 ^d^	262.16 ± 8.49 ^b^	323.13 ± 3.26 ^a^	259.03 ± 4.48 ^b^	205.58 ± 4.10 ^c^	206.60 ± 2.95 ^c^	205.66 ± 0.50 ^c^
Adhesiveness (g·s)	−61.21 ± 1.28 ^a^	−66.60 ± 1.46 ^b^	−68.65 ± 3.17 ^b^	−73.90 ± 2.73 ^c^	−75.42 ± 0.90 ^c^	−75.42 ± 0.90 ^c^	−80.25 ± 0.34 ^d^
Elasticity	0.92 ± 0.00 ^b^	0.97 ± 0.01 ^a^	0.97 ± 0.01 ^a^	0.93 ± 0.01 ^b^	0.87 ± 0.02 ^e^	0.88 ± 0.01 ^cd^	0.91 ± 0.01 ^bc^
Cohesion	0.83 ± 0.02 ^cd^	0.86 ± 0.00 ^ab^	0.89 ± 0.01 ^a^	0.86 ± 0.00 ^bc^	0.81 ± 0.00 ^d^	0.88 ± 0.01 ^ab^	0.76 ± 0.03 ^e^
Adhesive	154.12 ± 1.43 ^f^	241.24 ± 3.84 ^b^	287.08 ± 3.68 ^a^	213.50 ± 2.93 ^c^	169.44 ± 0.72 ^e^	179.84 ± 0.80 ^d^	170.80 ± 4.31 ^e^
Chewiness	138.83 ± 0.25 ^f^	227.99 ± 2.04 ^b^	274.47 ± 0.51 ^a^	192.26 ± 8.07 ^c^	146.72 ± 3.31 ^e^	162.85 ± 0.49 ^d^	165.44 ± 0.04 ^d^
Recovery	0.37 ± 0.01 ^bc^	0.37 ± 0.00 ^bc^	0.39 ± 0.00 ^b^	0.36 ± 0.01 ^c^	0.33 ± 0.00 ^d^	0.37 ± 0.01 ^c^	0.41 ± 0.00 ^a^

The values marked with different lowercase letters in each line have significant differences (*p* < 0.05).

## Data Availability

The original contributions presented in this study are included in the article/Supplementary Material. Further inquiries can be directed to the corresponding author.

## Supplementary Materials

The following supporting information can be downloaded at: https://www.mdpi.com/article/10.3390/foods14040626/s1, Table S1: Content and description of sensory evaluation of rice.

## Abbreviations

The following abbreviations are used in this manuscript:

SST	Superheated steam treatment
SEM	Scanning electron microscopy
DSC	Differential scanning calorimetry
FTIR	Fourier transform infrared spectroscopy
SAXS	Small-angle X-ray scattering
TPA	Texture profile analysis
