# Supplementary material for: Effect of Superheated Steam Treatment on Rice Quality, Structure, and Physicochemical Properties of Starch
Source: Foods. 2025 Feb 13;14(4):626. doi: 10.3390/foods14040626 (PMC11854516; doi:10.3390/foods14040626)
Supplement: Supplementary file 1 [file foods-14-00626-s001.zip › foods-3440142-supplementary.pdf]

Supplementary materials

Table S1. Content and description of sensory evaluation of rice

Primary indicators Score	Secondary indicators Score	Specific characteristic description: score
Smell (20 points)	/	Rich typical rice aroma: 18-20 points
		Clear typical rice aroma : 15-17 points
		Not obvious typical rice aroma : 12-14 points
		No aroma, but no unpleasant smell: 7-12 points
		Unpleasant smell: 0-6 points
Appearance structure (20 points)	Color (7 points)	Pure white: 6-7 points
		Normal: 4-5 points
		Yellow or gray: 0-3 points
	Gloss (8 points)	Clear luster: 7-8 points
		Slight luster: 5-6 points
		No luster: 0-4 points
	Integrity of rice grains (5 points)	Complete: 4-5 points
		Almost complete: 3 points
		Broken: 0-2 points
Palatability (30 points)	Viscosity (10 points)	Smooth, not stick to the teeth: 8-10 points
		Not easy to stick to the teeth: 6-7 points
		Easy to stick to the teeth, or no adhesiveness: 0-5 points
	Elasticity (10 points)	Chewy texture: 8-10 points
		Slight chewiness: 6-7 points
		Loose, hard: 0-5 points
	Hardness (10 points)	Moderate soft and hard： 8-10 points
		Slightly hard or soft: 6-7 points
		Too hard or soft: 0-5 points
Taste (25 points)	/	Rich fragrance and sweetness: 22-25 points
		A light rice fragrance and sweetness: 18-21 points
		No rice fragrance and sweetness, but no unpleasant smell: 16-17 points
		No rice fragrance and sweetness, unpleasant smell: 0-15 points
Cold rice texture (5 points)	/	Moderate hard: 4-5 points
		Slightly harder: 2-3 points
		Too hard: 0-1 points
